# The molecular basis of variable phenotypic severity among common missense mutations causing Rett syndrome

**DOI:** 10.1093/hmg/ddv496

**Published:** 2015-12-08

**Authors:** Kyla Brown, Jim Selfridge, Sabine Lagger, John Connelly, Dina De Sousa, Alastair Kerr, Shaun Webb, Jacky Guy, Cara Merusi, Martha V. Koerner, Adrian Bird

**Affiliations:** Wellcome Trust Centre for Cell Biology, University of Edinburgh, Michael Swann Building, Max Born Crescent, EdinburghEH9 3BF, UK

## Abstract

Rett syndrome is caused by mutations in the X-linked *MECP2* gene, which encodes a chromosomal protein that binds to methylated DNA. Mouse models mirror the human disorder and therefore allow investigation of phenotypes at a molecular level. We describe an *Mecp2* allelic series representing the three most common missense Rett syndrome (RTT) mutations, including first reports of *Mecp2*[R133C] and *Mecp2*[T158M] knock-in mice, in addition to *Mecp2*[R306C] mutant mice. Together these three alleles comprise ∼25% of all RTT mutations in humans, but they vary significantly in average severity. This spectrum is mimicked in the mouse models; R133C being least severe, T158M most severe and R306C of intermediate severity. Both R133C and T158M mutations cause compound phenotypes at the molecular level, combining compromised DNA binding with reduced stability, the destabilizing effect of T158M being more severe. Our findings contradict the hypothesis that the R133C mutation exclusively abolishes binding to hydroxymethylated DNA, as interactions with DNA containing methyl-CG, methyl-CA and hydroxymethyl-CA are all reduced *in vivo*. We find that MeCP2[T158M] is significantly less stable than MeCP2[R133C], which may account for the divergent clinical impact of the mutations. Overall, this allelic series recapitulates human RTT severity, reveals compound molecular aetiologies and provides a valuable resource in the search for personalized therapeutic interventions.

## Introduction

Mutations in the X-linked *MECP2* gene are implicated in several human disorders characterized by developmental delay and intellectual disability, including Rett syndrome (RTT) (1) and *MECP2* duplication syndrome (2). RTT is a condition with postnatal onset that predominantly affects girls, as males fail to survive beyond infancy. Animal models have proved useful for improving our understanding of MeCP2 function and for explaining in molecular terms the origin of the RTT phenotype. The first mouse models were simple loss-of-function alleles caused by gross deletion of most of the coding sequence (3,4), but knock-in mutations corresponding to specific RTT-causing mutations (5,6) offer the opportunity for deeper understanding. Of particular interest are missense RTT mutations leading to the substitution of a single amino acid, as these pinpoint critical regions of the protein that cannot be deduced from frameshift and nonsense mutations, or mutations that affect splicing (7). Importantly, human and mouse MeCP2 are highly homologous proteins, being ∼95% identical at the amino acid level across ∼500 amino acids. This stringent functional conservation over evolutionary time makes it likely that mutations in the mouse gene provide appropriate models for determining the molecular aetiology of the human disorders.

In this study, we generated knock-in mice for the three most common RTT missense mutations: T158M, R306C and R133C, which together account for ∼25% of all RTT cases. The most frequent of all RTT mutations is T158M (∼12% of cases), followed by R306C (∼9%) and R133C (∼5%) (8). Two of the three mutations (T158M and R133C) localize to the methyl-CpG binding domain (9), and previous *in vitro* studies suggest that both can adversely affect DNA binding (10–12). The R306C mutation is located within the recently defined NCoR/SMRT Interaction Domain (NID) (13). This mutation, like others close by, prevents MeCP2 from interacting with the NCoR/SMRT corepressor complex and inhibits transcriptional repression in cell-based assays. Although all three mutations result in ‘classical’ RTT symptoms, there is a clear difference between them when clinical severity rating scales from many patients are analysed (8,14). T158M is more severe than R306C on average. Least severe of the most frequent RTT mutations is R133C, which is more often associated with preservation of walking and/or speech (8). We sought to explain this clinical spectrum in molecular terms.

The R133C mutation has not previously been modelled in mice, but it has been proposed that its milder phenotype is due to retention of binding to 5-methylcytosine accompanied by loss of binding to hydroxymethylcytosine (hmC) (15). Mice with the T158A RTT mutation were characterized (6), but the much more common T158M RTT allele has not yet been reported. The R306C mutation has been shown to cause Rett-like phenotypes in knock-in mice (13), and mice bearing an integrated mutant transgene have been comprehensively phenotyped (16). Here, we focus on the comparative phenotypes caused by these three mutants at molecular, cytogenetic and behavioural levels. This was achieved by comparing lines of male mice expressing MeCP2 variants from the endogenous *Mecp2* locus as fusions with the reporter protein enhanced green fluorescent protein (EGFP), including wild type (WT). We find that these RTT mutations recapitulate the severity spectrum of human RTT, and they offer a molecular explanation for this phenotypic range. In particular, the R133C mutant protein has a reduced affinity for all known MeCP2 target sequences, including methyl-CG (mCG) and is significantly reduced in abundance. We conclude that the RTT-like phenotype cannot be exclusively attributable to loss of hmC binding by this mutant protein as previously proposed (15). The greater severity of the T158M mutation is explained by more extreme destabilization of the protein, coupled with reduced affinity for modified DNA. This finding mirrors that reported previously for a less frequent allele, T158A, affecting the same amino acid (6). R306C, which has lost the ability to interact with the NCoR/SMRT co-repressor, persists at WT levels and largely retains WT chromatin binding characteristics. This *Mecp2* allelic series allows resolution of the compound phenotypes underlying these causes of RTT. In addition, the study supports available prognostic information regarding Rett syndrome and provides a resource in the search for individualized therapeutic approaches.

## Results

### WT MeCP2-GFP mice are essentially phenotypically WT

**Figure 1.**
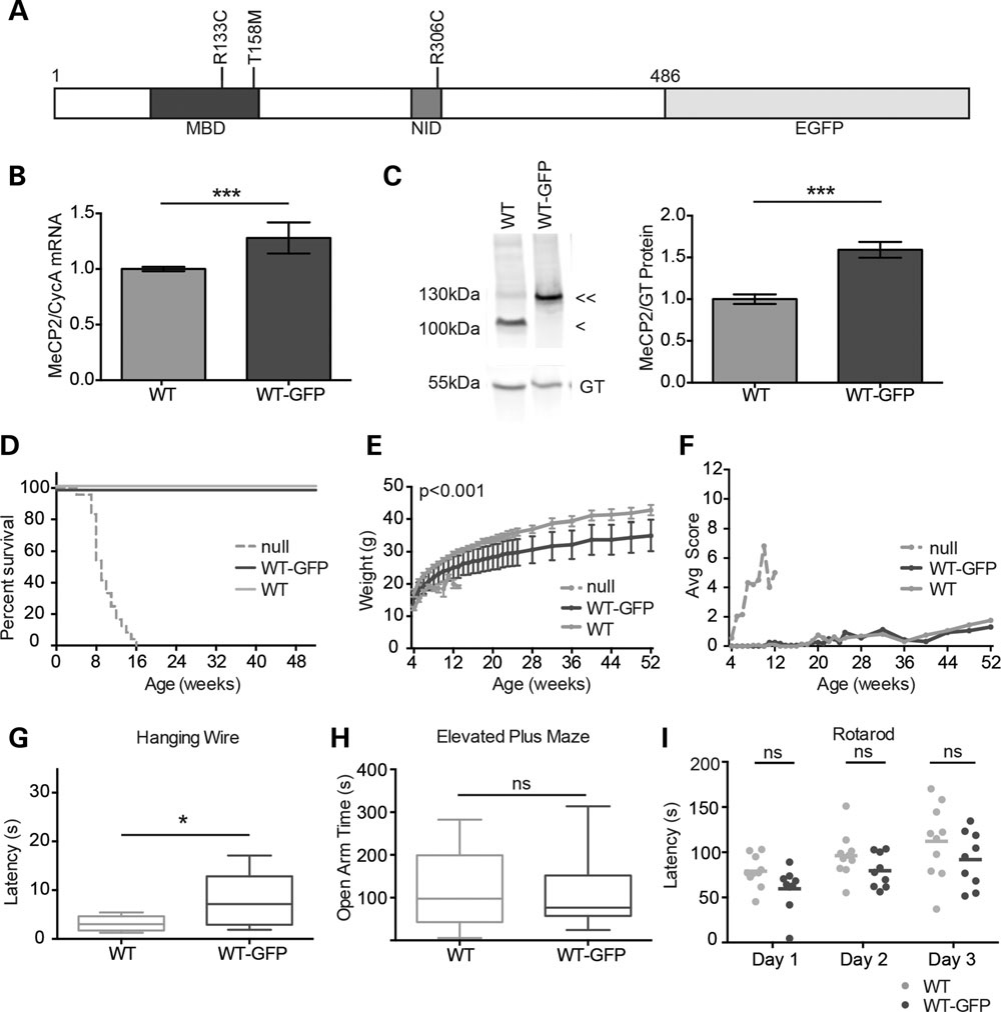
*WT-GFP* mice show minimal overt phenotype. (**A**) Schematic representation of MeCP2 with an EGFP tag. Missense mutations analysed in this study (T158M, R133C and R306C) are shown in relation to the MBD and NID. (**B**) Levels (mean ± SD) of *Mecp2* transcripts in *WT-GFP* mouse brain (*n* = 9) compared with *WT* littermates (*n* = 9), expressed relative to *Cyclophilin A* transcript (CycA). (**C**) Representative western blot and quantification comparing MeCP2 protein abundance in *WT-GFP* mouse brain (double arrow-head, *n* = 6) versus *WT* littermates (single arrow-head, *n* = 6). Gamma tubulin (GT) served as an internal control. Mean ± SEM plotted. (**D**) The Kaplan–Meyer plots showing survival of *WT-GFP* mice (*n* = 8) compared with their *WT* littermates (*n* = 8) and *Mecp2*-null mice [Ref. (18)] (*n* = 24). (**E**) Growth curve showing average weight of *WT-GFP* mice (*n* = 8) compared with their *WT* littermates (*n* = 8). Using repeated measures ANOVA, the difference was consistent and significant (*P* < 0.001) over time, but at any single time point, the difference was not significant. (**F**) Phenotypic scoring (see the text) of *WT-GFP* or *WT* mice. For comparison, Mecp2-null scoring is shown. (**G**–**I**) *WT-GFP* mice (*n* = 9) and their *WT* littermates (*n* = 10) were compared using three behavioural tests: (G) the hanging-wire test, (H) the elevated plus maze, and (I) the accelerating rotarod showing individual mean latencies (dots) and cohort mean latency (line) for each of three days of trials. Statistical analysis took all trials into account. Statistical tests were unpaired two-tailed *t*-test (B, C and E) and the Kolmogorov–Smirnov test (G–I). All behavioural paradigms were conducted on animals aged 8–10 weeks, and biochemical analyses were conducted using tissues from adults aged 6–12 weeks. Asterisks denote the following *P* values: *P < 0.05, **P < 0.01 and ***P < 0.001.

We generated an allelic series in which endogenous *Mecp2* or mutant *Mecp2* genes were fused in frame with EGFP at their C-termini (Fig. 1A; Supplementary Material, Fig. S1). Mice expressing knock-in WT *MeCP2-EGFP* fusion genes have been reported (13,17), but without extensive characterization. We initially looked for phenotypic defects due to the fusion of WT MeCP2 with EGFP by monitoring male mice at the molecular and whole organism levels. Analysis of hemizygous males has the advantage that phenotypic severity is not influenced by the pattern of X chromosome inactivation, and so severity of individual mutations can be assessed in an unbiased fashion. Quantitative polymerase chain reaction (PCR) and western blots indicated that both mRNA and protein products of the *Mecp2-GFP* gene (*WT-GFP*) were expressed in brain, though at somewhat higher levels than the endogenous *Mecp2* gene (*WT*) (Fig. 1B and C). Quantitative western blots suggested that the level of WT-GFP is ∼1.6-fold higher than in untagged WT littermates. At the whole organism level, we analysed cohorts of *WT-GFP* mice back-crossed for four generations to give a genetic background that is ∼94% C57BL/6J. *WT-GFP* knock-in mice were fertile and showed normal survival but tended to be smaller than WT littermates (Fig. 1D and E). Cohorts were monitored using a phenotypic scoring methodology that records breathing, tremor, gait, hindlimb clasping, mobility and general condition (18). This series of observational tests has the advantage that it is not affected by learning and can therefore be performed weekly over long periods, giving reproducible results. Using this method, *WT-GFP* mice showed no significant phenotypic deterioration compared with *WT* littermates (Fig. 1F), reinforcing the view that, despite the presence of the EGFP tag, they are essentially WT.

To search for neurological phenotypes in more detail, we subjected *WT-GFP* mice to a series of motor coordination and behavioural tests (Fig. 1G–I). Performance on the elevated plus maze was indistinguishable from *WT* and on the accelerating rotarod was also not significantly different from *WT* littermates. The hanging-wire test showed a weak but reproducible reduction in the ability to engage hindlimbs with the wire. We noted in addition that there was a trend towards a mild reduction in weight and a trend towards defective rotarod performance, but neither achieved significance. These very weak phenotypic effects may be attributable to the over-expression of MeCP2-GFP relative to untagged protein. Taking the findings together, however, we conclude that the addition of the C-terminal EGFP epitope and the moderate over-expression of the protein have minimal phenotypic consequences by these assays.

### Allelic series of RTT missense mutations recapitulates severity in humans

**Figure 2.**
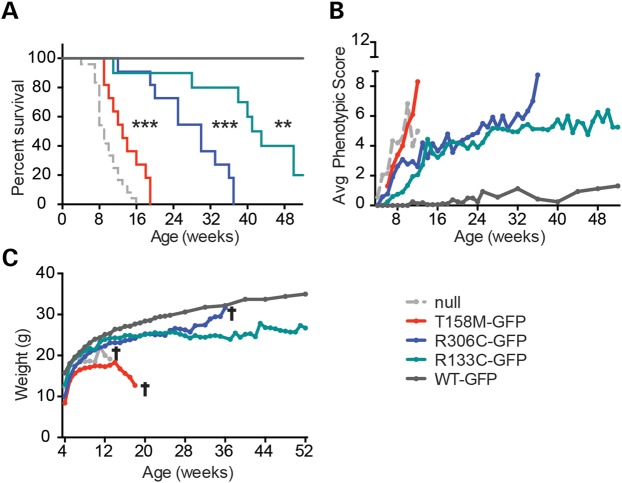
The *Mecp2-GFP* allelic series mimics the clinical severity of equivalent human mutations. (**A**) The Kaplan–Meyer plots showing reduced survival of *T158M-GFP* (red), *R306C-GFP* (blue) and *R133C-GFP* (green) mutants in comparison with *WT-GFP* (dark grey) and *Mecp2*-null (light grey) mice (18). Statistical significance is denoted as follows: *P < 0.05, **P < 0.01 and ***P < 0.001 (Mantel-Cox test) and calculated in comparison with the *WT-GFP* mice. (**B**) Graph showing average phenotypic score for each cohort over time. (**C**) Graph showing average weight over time. Cohorts comprised *WT-GFP* (*n* = 8), *R133C-GFP* (*n* = 10), *R306C-GFP* (*n* = 11), *T158M-GFP* (*n* = 11) and *Mecp2*-null mice (*n* = 12–24). A cross indicates that all mice in the cohort had been culled by this time point due to severity of the RTT-like phenotype.

Using the same knock-in technology, we generated the following mouse lines: *Mecp2*[T158M]^EGFP^, *Mecp2*[R306C]^EGFP^ and *Mecp2*[R133C]^EGFP^, referred to as *T158M-GFP*, *R306C-GFP* and *R133C-GFP*, respectively. Both MeCP2 isoforms, which differ only at their extreme N-termini, are affected by the knock-in. Each line was back-crossed to obtain a predominantly C57BL/6J genetic background equivalent to that of the *WT-GFP* mice (94%). Each of the mutants gave rise to males that exhibited overt phenotypic defects from ∼6 weeks of age (see below), but their survival profiles were very different (Fig. 2A). *T158M-GFP* male survival was slightly prolonged compared with *Mecp2*-null mice from previous analyses (18) (median survival 13 weeks compared with 9 weeks), whereas *R306C-GFP* males survived significantly longer (median = 30 weeks). *R133C-GFP* males were most mildly affected, with a median survival of 42 weeks.

**Figure 3.**
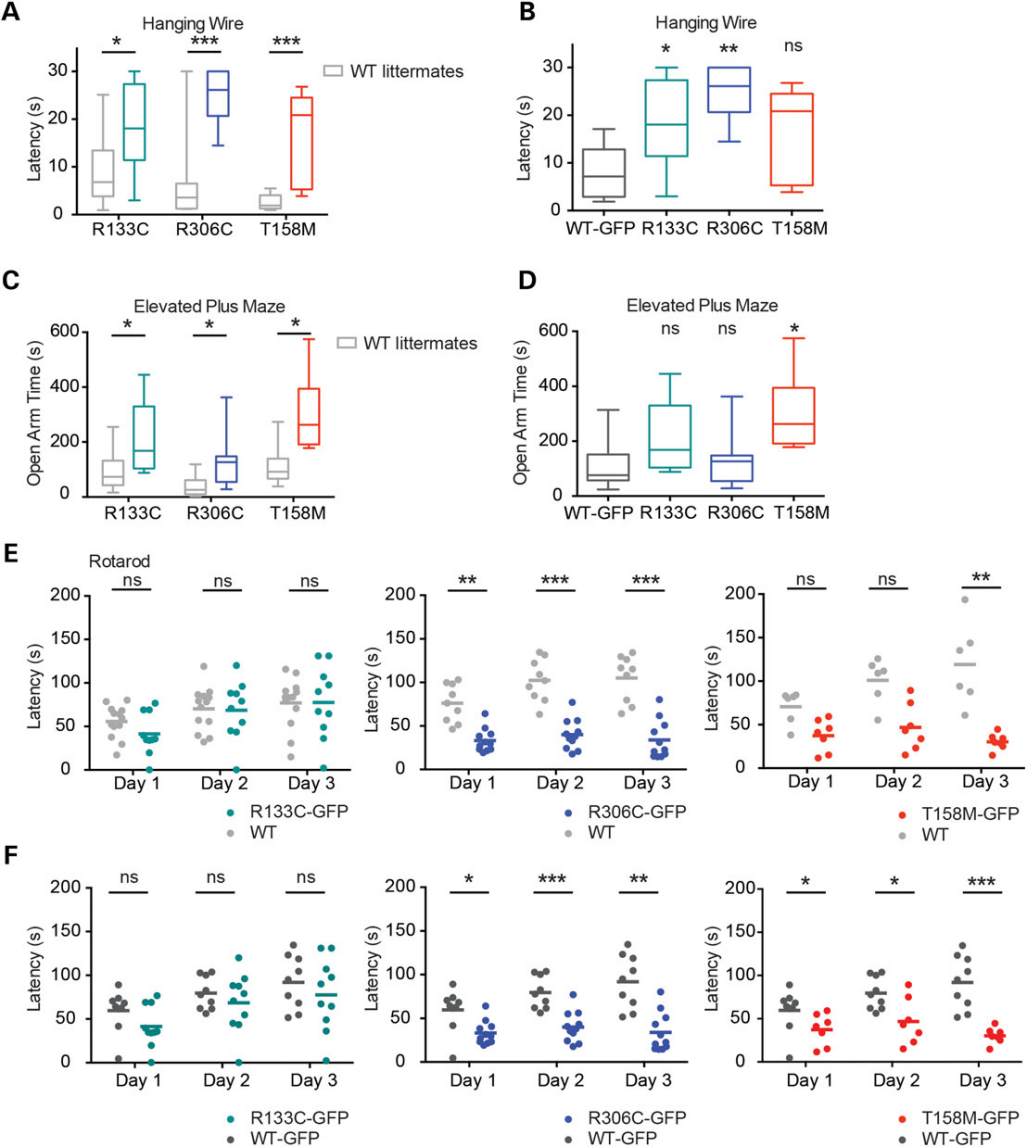
Behavioural analysis of the *Mecp2-GFP* allelic series indicates that *R133C-GFP* mice are less severely affected than *R306C-GFP* and *T158M-GFP*. Mutant males and *WT* male littermates underwent behavioural analysis as in Figure 1. (**A**) Hanging-wire test, (**C**) elevated plus maze and (**E**) accelerating rotarod. *R133C-GFP* (*n* = 10, green) plus *WT* littermates (*n* = 13, grey); *R306C-GFP* (*n* = 10–11, blue) plus *WT* littermates (*n* = 9, grey); *T158M-GFP (n* = 7, red) plus *WT* littermates (*n* = 6, grey). **B**, **D** and **F** show comparisons between mutant males and *WT-GFP* males in the same series of tests. (**E** and F) Graphs showing mean time to descent for individuals (dots) and cohorts (lines) for each trial day. Statistical analysis took all trials into account, and significance is denoted as follows: *P < 0.05, **P < 0.01 and ***P < 0.001 (Kolmogorov–Smirnov test). All behavioural paradigms were conducted on animals aged 8–10 weeks.

Phenotypic scoring of *T158M-GFP*, *R306C-GFP* and *R133C-GFP* mice matched survival curves and provided detail regarding progression (Fig. 2B). *T158M-GFP* male mice acquired a range of phenotypic traits rapidly over a few weeks, whereas *R306C-GFP* mice, after an initially rapid onset, remained phenotypically relatively stable until 33 weeks, at which time phenotypes of survivors became more severe. Interestingly, this late inflection point coincides with an age identified previously as being particularly sensitive to MeCP2 deficiency (19). *R133C-GFP* males showed a more gradual build-up of phenotypic defects compared with either *R306C-GFP* or *T158M-GFP*. Weight profiles revealed that all the mutants were lighter than *WT-GFP* animals of equivalent age (Fig. 2C). Cohorts of the allelic series underwent motor coordination and behavioural testing at 8–10 weeks of age at which time *T158M-GFP*, *R306C-GFP* and *R133C-GFP* mice scored 5.4, 2.8 and 1.4, respectively, using the observational scoring method (see Fig. 2B). None of the mutants performed as well as their corresponding *WT* littermates on the hanging wire and elevated plus maze (Fig. 3A and C). *T158M-GFP* and *R306C-GFP* were also significantly compromised as assayed by the 3-day accelerating rotarod-learning paradigm (Fig. 3E), but *R133C-GFP* mice showed no detectable defects compared with WT in this test.

In these analyses, WT littermates lacked the GFP tag on endogenous MeCP2, but as shown in Figure 1, the presence of this tag has minimal phenotypic consequences. We confirmed that the presence of C-terminal GFP was not contributing to phenotype by comparing the performance of mutant-GFP mice with age-matched WT-GFP mice in the same tests (Fig. 3B, D and F). Comparison between litters of different lines in this way is less well controlled, as illustrated by the variability of WT test scores in Figure 3A and C. Nevertheless, we observed that both R133C and R306C mice performed significantly less well than WT-GFP in the hanging-wire and rotarod tests, while performance of T158M mice in both elevated plus maze and rotarod was significantly inferior. In cases where differences between mutant and WT-GFP mice did not reach statistical significance (R133C and R306C in the elevated plus maze, and T158M on the hanging-wire test), the data showed trends towards defective performance that matched those detected in the comparison with littermates. The behavioural analyses therefore confirm phenotypic scoring and survival data in showing that the Rett missense mutations are responsible for the observed phenotypes.

### Heterozygous females show RTT-like phenotypes

Rett syndrome affects females who are heterozygous for these *MECP2* mutations and consequently mosaic for expression of WT or mutant alleles, due to X-chromosome inactivation. In mice, heterozygotes for the null allele show a delayed-onset phenotype that is usually detectable between 4 and 12 months of age. We compared phenotypic scores of heterozygotes for each mutation over ∼12 months. *WT-GFP* female heterozygotes were not scored in this fashion as they invariably displayed no signs of disease up to at least 1 year. All had RTT-like symptoms, with *R133C-GFP* and *R306C-GFP* being less severe than *T158M-GFP* (Supplementary Material, Fig. S2). In this respect, also the phenotypes of these frequent RTT mutations in mice reflect the spectrum of human severity.

### MeCP2 R133C-GFP and T158M-GFP show reductions in both abundance and chromatin binding in neurons

**Figure 4.**
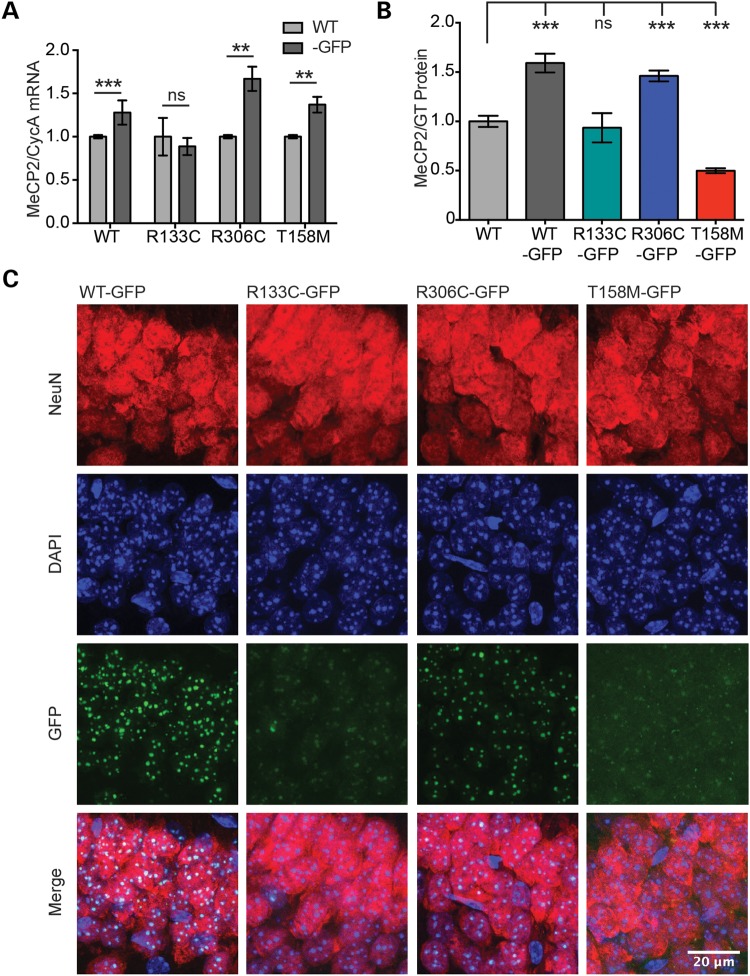
R133C-GFP and T158M-GFP are less abundant than WT-GFP and have an abnormal pattern of sub-nuclear localization. (**A**) Graph showing level of *Mecp2* transcript normalized to *Cyclophilin A* in mutant male mouse brains relative to *WT* littermates (mean ± SD). *WT-GFP, n* = 9; *R133C-GFP*, *n* = 4; *R306C-GFP*, *n* = 3; *T158M-GFP*, *n* = 3. (**B**) Quantification of western blots showing levels of WT-GFP, R306C-GFP, R133C-GFP and T158M-GFP protein in male mouse brain, relative to WT (mean ± SEM). GT served as an internal control; *WT*, *n* = 6; *WT-GFP*, *n* = 6; *R133C-GFP*, *n* = 3; *R306C-GFP*, *n* = 4; *T158M-GFP*, *n* = 6. (**C**) Representative images of the CA1 region of the hippocampus in adult male mice from the allelic series. Slices were imaged using the same confocal settings. Immunofluorescence was performed with DAPI (blue) and anti-NeuN (red). MeCP2 was imaged by virtue of its EGFP tag. R133C-GFP and T158M-GFP show mixed punctate and diffuse sub-nuclear localization. Scale bar = 20 μm. Statistical significance is denoted as follows: *P < 0.05, **P < 0.01 and ***P < 0.001 (two-tailed unpaired *t*-test). All biochemical analyses were conducted using tissues from adults aged 6–12 weeks.

The matching spectrum of severity between humans and mice with respect to *T158M-GFP*, *R306C-GFP* and *R133C-GFP* mutations provides validation that the mouse models are an appropriate system to investigate the mechanism by which these mutations cause RTT in humans. We used cell biological and biochemical techniques to understand the reasons for compromised MeCP2 function. At the level of mRNA, expression of the *Mecp2*-*GFP* knock-in alleles in brain was somewhat elevated compared with the native *Mecp2* gene, except in the case of *R133C-GFP* (Fig. 4A). In agreement with the mRNA analysis, WT-GFP and R306C-GFP proteins were 1.5-fold more abundant than native MeCP2 in the brain, with the expression of R306C-GFP modestly reduced relative to WT-GFP (Fig. 4B). In contrast, R133C-GFP protein was reduced to ∼55%, and T158M-GFP was present at only ∼30% of WT-GFP by western blotting (Fig. 4B and Supplementary Material, Fig. S3A) and fluorescence-activated cell sorting (Supplementary Material, Fig. S3B). Abundance was therefore equivalent to approximately 95 and 50% of untagged MeCP2 for R133C-GFP and T158M-GFP, respectively. Reduced stability of T158A, a distinct allele of T158, was reported previously (6), making it likely that this is an intrinsic property of MeCP2 lacking the hydrogen-bonding capability of threonine at this position (20). To determine whether the more subtle reduction in R133C-GFP seen in the brains is specific to this particular mouse line or is a reproducible property of the mutant protein, we analysed embryonic stem cell-derived neurons in culture using an independent knock-in clone. Again R133C-GFP was present at approximately half the level of WT-GFP, suggesting that the mutation itself is responsible for the deficiency, probably due to compromised RNA or protein stability (Supplementary Material, Fig. S3C). This relationship was also seen with an alternative *WT-GFP* clone (data not shown).

Taking advantage of GFP immunofluorescence, we examined localization of the different MeCP2 variants in hippocampal sections of hemizygous male mouse brains (Fig. 4C). The intensities of nuclear EGFP immunofluorescence in brain sections confirmed qualitatively that in comparison with WT-GFP protein, R306C-GFP expression was similar, R133C-GFP expression was moderately reduced and T158M-GFP was severely reduced in the brain. We focussed on sub-nuclear localization of the MeCP2 variants, as about half of all 5-methylcytosine in the mouse genome is concentrated in pericentromeric foci due to the abundance and CG richness of mouse satellite DNA sequences. Native MeCP2 localized to pericentromeric foci in a DNA methylation-dependent manner (21) as did WT-GFP and R306C-GFP fusion proteins. R133C-GFP and T158M-GFP, however, showed a more diffuse distribution throughout the nucleoplasm coupled with reduced heterochromatic localization. Diffuse nuclear staining in comparison with WT-GFP and R306C-GFP mutant proteins was more apparent at higher confocal laser intensity, which showed background nuclear EGFP fluorescence in addition to heterochromatic localization (Supplementary Material, Fig. S3D). This effect was more severe for T158M-GFP than for R133C-GFP. An equivalent pattern was also seen in embryonic stem cell-derived neurons in culture (Supplementary Material, Fig. S3E). The results suggest compromised binding to mCG sites by both these mutants, as explored below.

### Compromised binding of MeCP2[R133C] and MeCP2[T158M] to modified DNA *in vivo* and *in vitro*

**Figure 5.**
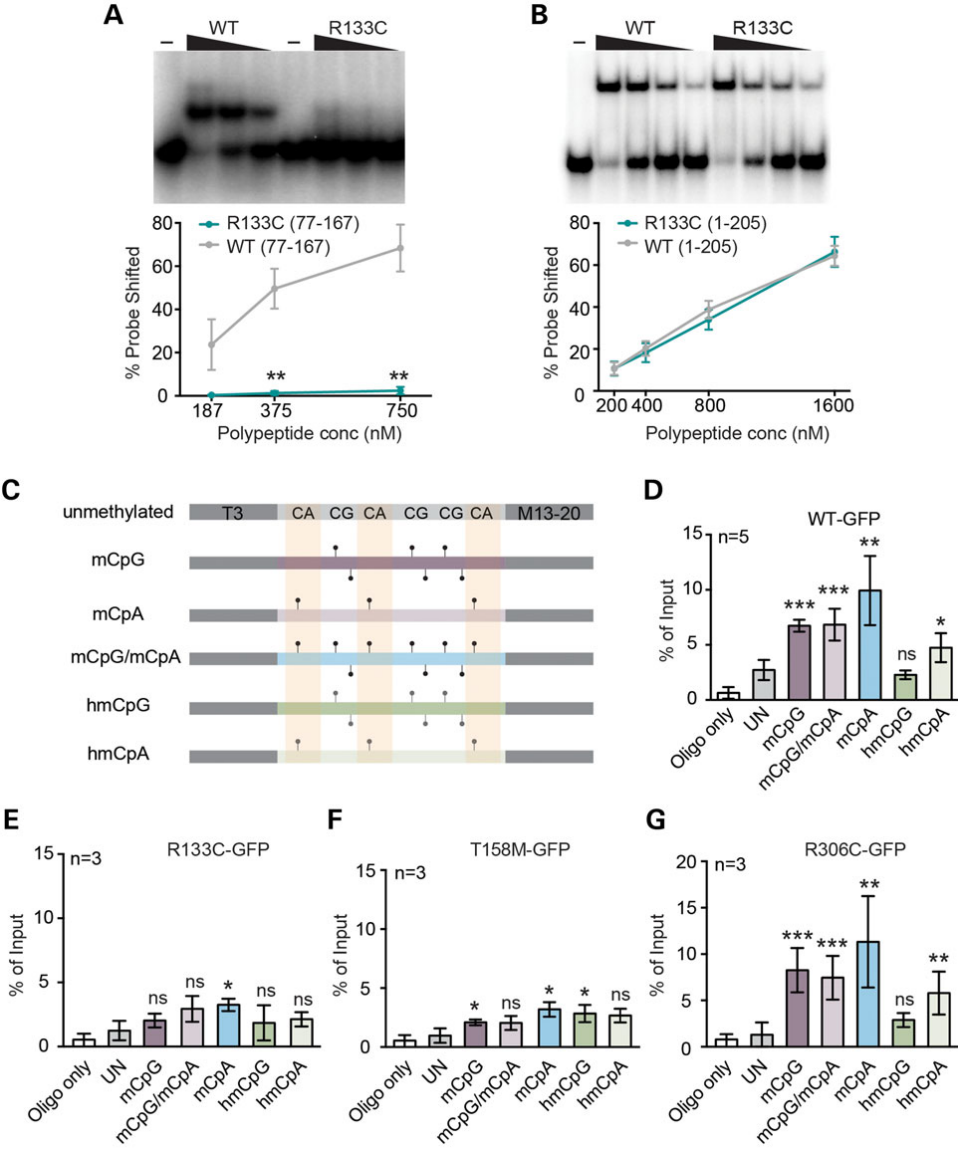
Defective binding of MeCP2[R133C] and T158M to modified DNA *in vitro* and *in vivo*. Representative gels and quantification of EMSAs measuring MeCP2 peptide binding to mCG probes using (**A**) aa77-167 or (**B**) aa1-205 fragments of MeCP2 with (green) or without (grey) the R133C mutation. Mean percentage of probe shifted ± SEM is plotted from three replicated experiments. Triangle represents decreasing peptide concentration; –, no peptide. Statistical significance is denoted as: *P < 0.05, **P < 0.01 and ***P < 0.001 (unpaired two-tailed *t*-test). (**C**) ChIP oligonucleotide duplex probes containing symmetrical mCG or hmCG, or asymmetrical mCA or hmCA sites or both mCA and mCG sites together. Control DNA lacks all modifications. (**D**–**G**) Results of five independent transient transfection experiments followed by ChIP of MeCP2-associated probe oligonucleotide fragments. Cells transiently expressed WT-GFP, R133C-GFP, T158M-GFP and R306C-GFP, respectively. Mean % input ± SD plotted (*n* ≥ 3).

Published evidence regarding the DNA binding affinity of R133C protein is inconsistent. South-western (10,22) and EMSA (10,11,22,23) analyses revealed reduced binding of R133C to methylated DNA. Expression of R133C protein from an integrated transgene in embryonic stem cells also revealed reduced specificity for methylated DNA (24). On the other hand, surface plasmon resonance indicated that the affinity of MeCP2 for CG-methylated DNA is unaffected by the R133C mutation, whereas binding to hydroxymethylated DNA is greatly reduced (15). In a transfection assay, over-expressed R133C-GFP localized normally to heterochromatin (12,24,25), although the mobility in a FRAP assay was somewhat increased, compatible with a modest reduction in affinity for DNA (25,26). In view of the somewhat variable observations, we repeated EMSAs using either the methylated DNA binding domain (MBD) alone (amino acids 77–167) or a larger N-terminal fragment of MeCP2 (amino acids 1–205). Interestingly, these two polypeptides showed very different DNA binding affinities when carrying the R133C mutation. The 77–167 fragment of R133C, which corresponds to the MBD alone, lost the ability to form a stable complex with methylated DNA, whereas 1–205[R133C] was essentially WT by an equivalent assay (Fig. 5A and B). These opposing findings with a common probe and under the same conditions recapitulate the spectrum of *in vivo* and *in vitro* findings in the literature for this mutant, and they leave open the question of whether this defect has any impact on chromatin association in living cells.

To address this issue, we used a cellular assay in which the various forms of MeCP2 are over-expressed transiently at equivalent levels in the presence of double-stranded oligonucleotides bearing specific modifications of cytosine (S. Lagger *et al*., manuscript in preparation). The method has the advantage that it detects binding of full-length protein in living cells while allowing the modification status of the target DNA to be precisely defined. In this way, we could test binding not only to mCG, but also to methyl-CA (mCA) and hydroxymethyl-CA (hmCA), which are all implicated as target sequences for MeCP2 (27). Double-stranded oligonucleotides containing either three symmetrically methylated mCGs, three hmCGs or both mCA and mCG modifications (Fig. 5C) were transiently transfected into human embryonic kidney (HEK293) cells that transiently expressed WT MeCP2-GFP (WT-GFP) or mutant MeCP2-GFP from plasmid constructs. Unmodified oligonucleotides of the same sequence served as controls. Recovery of the target DNA by immunoprecipitation with anti-MeCP2 antibodies was monitored by quantitative PCR (qPCR). The results showed preferential binding of WT-GFP protein to DNA containing mCA, mCG and, less efficiently, hmCA compared with non-methylated DNA, confirming the specificity of the method (Fig. 5D). R306C-GFP, whose missense mutation is outside the DNA binding domain bound to methylated DNA indistinguishably from WT (Fig. 5G). Mutant R133C-GFP and T158M-GFP proteins, in contrast, showed greatly impaired binding to all modified oligonucleotides (Fig. 5E and F). Importantly, protein expression levels were closely similar between experiments (Supplementary Material, Fig. S4), and therefore the effects of the mutation on protein stability as seen in mouse brain do not affect these results. Of particular note, the R133C mutation significantly compromises the interaction between MeCP2 and all modified oligonucleotides *in vivo*, including mCG.

**Figure 6.**
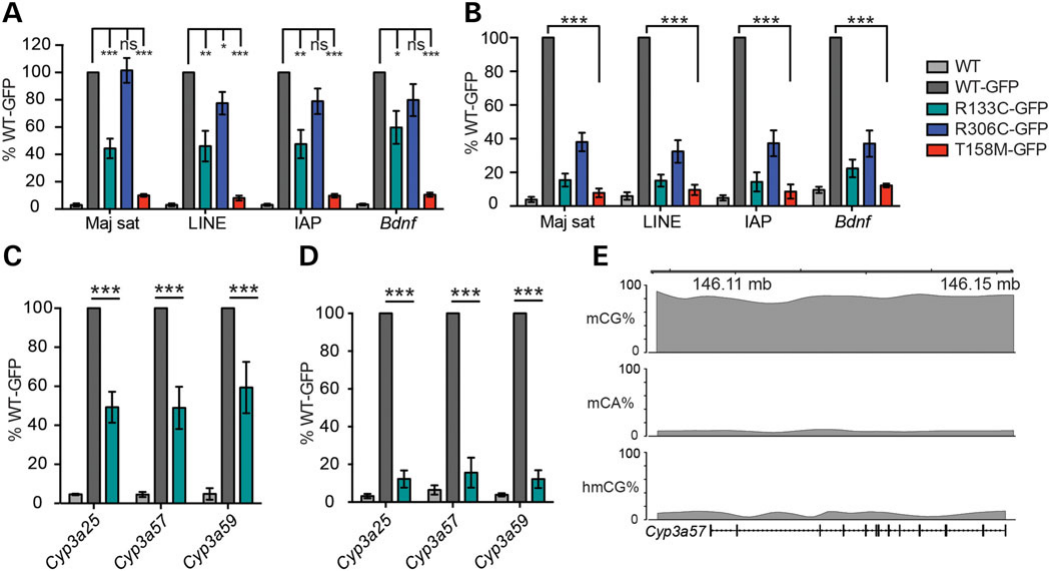
Chromatin immunoprecipitation reveals abnormal binding of R133C-GFP to mCG-rich repetitive sequences and genes in mouse brain. (**A**) ChIP-qPCR of WT-MeCP2 (lacking a GFP tag) and knocked-in WT-GFP, R133C-GFP, R306C-GFP and T158M-GFP in the adult male mouse brain. Primers amplify, respectively, major satellite, LINE and IAP transposable elements and the *Bdnf* gene locus. The antibody was against GFP and therefore does not precipitate untagged WT MeCP2 (*n* ≥ 3). (**B**) Similar assay to (A) but with a lower and therefore more stringent cross-linking temperature (*n* = 4). (**C**) Column plot showing binding of R133C-GFP at the mCG-rich cytochrome p450 locus by GFP ChIP (*n* = 3). (**D**) The same assay as (C) but using a more stringent cross-linking temperature. Plots are expressed as % WT-GFP value. Error bars represent ± SEM, and statistical significance is denoted as: *P < 0.05, **P < 0.01 and ***P < 0.001 (unpaired, two-tailed *t*-test). Each replicate experiment used tissue from a separate animal. (**E**) Schematic of the *Cyp3a57* locus depicting cytosine methylation context. All biochemical analyses were conducted using tissues from adults aged 6–12 weeks.

To examine the effects of mutations on MeCP2 binding in the native genome *in vivo*, we quantitatively analysed R133C-GFP and T158M-GFP binding in mouse brain by ChIP-qPCR using an anti-EGFP antibody. For comparison, we also analysed WT-GFP and R306C-GFP mutant proteins. The results showed that recovery of mouse satellite DNA, LINE1 elements, intracisternal A particles and the gene for brain-derived neurotrophic factor (*Bdnf*) from cross-linked brain chromatin by R133C-GFP was reduced to ∼45–60% of the WT-GFP level, whereas T158M-GFP was 5–10% of WT-GFP (Fig. 6A). Binding of R306C-GFP protein was not significantly different from WT-GFP. We were concerned that reduced binding of the two MBD mutants might reflect their low abundance rather than an effect on binding affinities *per se*. We therefore increased the stringency of cross-linking by reducing the fixation temperature on ice. Under these conditions, ChIP of the R133C-GFP protein was reduced to ∼15% of the WT-GFP level, which is significantly greater than the reduction in protein abundance of ∼50%. It was noticeable that under these stringent conditions binding by R306C-GFP was also compromised, as reported by others (16), although this was not seen using our standard cross-linking protocol (Fig. 6B). The ChIP results, together with the *in vitro* data, transfection experiments and immunofluorescence, suggest that both T158M-GFP and R133C-GFP mutant proteins have a significantly reduced affinity for chromatin.

As MeCP2 is proposed to bind to both mCA- and hmC-containing DNA (16,27), we considered the possibility that the reduced ChIP signal from *R133C-GFP* brain may be due to compromised binding to these sequences rather than to mCG. To test this, we probed immunoprecipitates for non-repetitive DNA sequences that contain mCG, but very low levels of mCA and hmCG. The ∼900 kb cytochrome P450 gene locus, which is transcriptionally silent in the brain, meets this requirement as whole-genome bisulphite and TAB sequencing show a high level of CG methylation (28), while the other proposed MeCP2 binding sites, hmC and mCH, are rare (Fig. 6E). Three of the genes from this locus, *Cyp3a25*, *Cyp3a57* and *Cyp3a59* represented this pattern of methylation and were selected for ChIP analysis. Again, recovery of these sequences by ChIP was reduced in *R133C-GFP* brain extracts, reinforcing the conclusion that mCG binding by R133C-GFP is significantly compromised (Fig. 6C and D).

### Modestly increased expression of long genes in the cerebellum of R133C-GFP mice

Previous studies have detected subtle changes in gene expression in the brains of mice that are *Mecp2*-null or carry RTT mutations (29). We compared gene expression in cerebellums of *R133C-GFP* and *WT-GFP* mice using expression microarrays, along with *WT* and *Mecp2*-null mice for reference. The results initially showed statistically significant differences at six individual loci in *R133C-GFP* cerebellum compared with *WT-GFP*, namely *Abhd1*, *Aplf*, *Dag1*, *Zfp428* (all down-regulated), *Mybpc3* and *Krt222* (*up-regulated*), but only two of these were reproduced by qPCR. In one case, the effect was very small. In the other case (*Abhd1*), the *R133C-GFP* expression was unchanged relative to *WT* or null comparators. In fact, the variance in expression was attributable to differences between WT-GFP cerebellum and the other tested tissues (Supplementary Material, Fig. S5A). We explored the data further by binning genes according to length to replicate recent studies showing that long genes are disproportionately up-regulated in *Mecp2* mutant brains (27,30). The results showed a small but sustained length-dependent increase in expression of longer genes in *R133C-GFP* versus *WT-GFP* cerebellum (Supplementary Material, Fig. S5B). In this study, the up-regulation of long genes was modest in the *Mecp2*-null versus *WT* cerebellum, which was in agreement with previous similar analyses of this brain region (27).

## Discussion

MeCP2 is identical between human and mouse throughout the great majority of its sequence, suggesting that almost all residues of the protein are essential for full function. Despite long-range conservation of the primary sequence across evolution, the distribution of missense mutations causing RTT is highly non-random (13). Most missense mutations are concentrated in the MBD and NID domains, disrupting the interaction with DNA and NCoR/SMRT, respectively (7). All of the most common RTT mutations, including nonsense truncations, potentially involve C to T transitions at CG sites and are therefore most likely caused by 5-methylcytosine deamination coupled with failure of repair (31). Their high frequency of occurrence therefore reflects elevated mutability. The most common missense mutation is T158M, which disrupts hydrogen-bonding integral to maintaining the configuration of an ASX turn (Asp156–Phe157–Thr158) and following ST motif (Thr158–Val159–Thr160–Gly161) in the C-terminal region of the MBD. This has been shown to destabilize MBD structure and reduce affinity for methylated DNA (20). Next most frequent is R306C, which is located in the NID and prevents the interaction of MeCP2 with the NCoR/SMRT co-repressor complexes (13). Least severe on average of the common RTT mutations is R133C, which mutates an arginine residue that in the X-ray structure makes hydrogen bond contact with one of the guanines in the symmetrical CG dyad (20). Individuals with this mutation more frequently retain some speech and the ability to walk (8,14). We found that phenotypic severity in mice matches the respective clinical severity of these mutations in humans, suggesting that the molecular basis of the resulting disorders is the same in the two species.

The effects on DNA binding of MeCP2[T158M] to DNA are somewhat inconsistently reported in the literature. Previous *in vitro* studies of full-length T158M protein showed either mild (10) or severe (22) impairment of mCG binding by South-western assay. EMSA analysis, using the MBD fragment, also revealed ambiguous findings. Reduced binding to methylated DNA (10,20,23) was seen in some instances but not others (11). Some of these discrepancies may be attributed to the positions of N- and C-terminal affinity purification tags (23). *In vivo* analysis of murine fibroblasts over-expressing full-length MeCP2[T158M] indicated defective binding to heterochromatic foci, and FRAP analysis showed greater mobility of MeCP2[T158M] compared with WT protein, indicating a less stable association with chromatin (25,26). Most published evidence therefore supports the view that the T158M mutation compromises binding between MeCP2 and methylated DNA. Our ChIP data based on transient transfection of cultured cells and brain confirm this defect in chromatin binding. These findings agree with an analysis of a different *Mecp2* allelic variant involving the same amino acid, T158A (6).

Molecular and cell biological assays allowed us to critically assess a recent hypothetical explanation for the milder phenotype in *R133C-GFP* mice. It was proposed that MeCP2[R133C] retains WT levels of binding to mCG *in vitro*, but has specifically lost the ability to interact with hydroxymethylated DNA (15). Our findings place this hypothesis in doubt as several lines of evidence show that mCG binding by MeCP2 is significantly compromised by this mutation: (i) immunofluorescence analysis in differentiated cultured neurons and in hippocampal sections showed diffuse nuclear staining with reduced localization to densely methylated heterochromatic foci; (ii) MBD DNA binding *in vitro* was defective by EMSA, in agreement with most previous reports (though a longer fragment of MeCP2 displayed normal binding); (iii) a cellular assay showed that full-length R133C-GFP binding to mCG was greatly reduced; and (iv) ChIP analysis of genomic regions where mCG was the only detectable modification showed greatly reduced binding by the mutant protein in the brain. The brain ChIP data are particularly compelling, as mouse satellite sequences and a gene cluster, both of which lack hmC and mCH, showed impaired binding *in vivo*. In addition to this loss of mCG binding, transfection experiments showed a significant reduction in MeCP2[R133C] binding to the alternative canonical binding sites mCA and hmCA.

**Figure 7.**
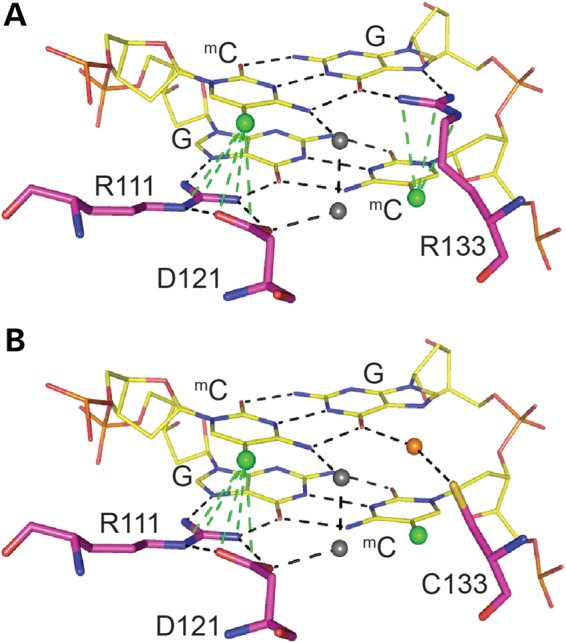
Predictive modelling of MeCP2 MBD binding to methylated DNA reveals a loss of specificity with the R133C mutation. (**A**) Predicted model of MBD binding to a methylated CpG pair. Arginine 111 and 133 make contact with the guanines and form hydrogen bonds with the methylcytosines of the CpG pair. Arginine 111 forms a hydrogen bond ‘clip’ with aspartic acid 121. (**B**) Predicted model of MBD[R133C] binding to a methylated CpG pair. The cysteine 133 interaction with guanine is now dependent on water molecules, and there is no significant interaction with the methylcytosine. (A and B) Hydrogen bonds are indicated by dotted lines, methyl groups by green balls, and water molecules by grey and orange balls.

Despite occupying a key position in the MBD of MeCP2, the R133C mutation results in a milder RTT phenotype and displays residual mCG binding. These observations can be reconciled by modelling the MeCP2–DNA interaction at a molecular level (Fig. 7). The X-ray structure shows that two arginines, R133 and R111, form equivalent hydrogen bonds with the two guanine residues located on opposite strands at the self-complementary dinucleotide sequence 5′CG (20) and also interact with 5-methylcytosine (Fig. 7A). Replacement of R111 by glycine causes RTT, as expected if DNA binding is severely disrupted. Substitution of R133 by cysteine might be expected to have an equally severe effect, but in fact residual DNA binding persists. A notable difference between R111 and R133 is that the side chain of the former adopts an all-trans extended configuration and its side-chain amino group is held in this position by aspartic acid 121 via a hydrogen bond ‘clip’. Loss of this interaction probably has structural consequences for the MBD as a whole. In contrast, the R133 side chain is relatively untethered, forming a single salt bridge with the carboxylate group of glutamate at position 137 (20). Replacement of R133 by the compact cysteine residue is unlikely to impact the integrity of the MBD, but due to loss of interactions with mC and G will reduce specificity. By placing a hypothetical water molecule in the major groove, it is possible to generate hydrogen bonding with G (Fig. 7B), which may account for the retention of weak DNA binding. We and others have previously reported that WT MeCP2 fails to interact with hmCG *in vitro* (27,32–34). As hmCG is by far the most abundant setting for hmC in the brain (28), it is unclear which hmC moieties might be targeted *in vivo*. Further work may be needed to define the full range of DNA binding specificities for both WT and mutant forms of MeCP2.

The R306C mutation occurs outside the MBD of MeCP2 and has no obvious effect on its stability, but may weakly affect chromatin binding (see also (16)). DNA binding impairment of this protein domain has not been characterized biochemically and was not detected in our cellular assay with full-length protein. It is possible that it is indirect, due for example to differences in extraction or altered association with partners. Further work is needed to address this issue. The two MBD mutations T158M and R133C, however, display both compromised stability and weaker DNA binding. Therefore, it is unlikely that a reduced affinity for chromatin alone underlies their clinical effects. In both cases, MeCP2 abundance was found to be lower than WT-GFP in knock-in mouse lines and when independent knock-in ESCs were differentiated into neurons in culture. R133C-GFP exhibited a less pronounced reduction in abundance (∼55%) in mice compared with T158M-GFP (∼30%). We conclude that the reduced abundance of T158M-GFP and R133C-GFP proteins is an intrinsic property conferred on the protein by the respective mutations. We speculate that the differing average severity associated with these two mutations is primarily due to their contrasting abundance. If both mutant proteins were present at the WT level, through pharmacological stabilization or over-expression, we speculate that they would exhibit similar pathologies at the milder end of the RTT spectrum.

The introduction of specific RTT mutations into mice confirms the value of this model system in studying the molecular basis of this condition. Generalizations are starting to emerge—for example, three missense mutations (T158A, T158M and R133C) have now been found to destabilize the protein and almost certainly make a significant contribution to the phenotype (this work and reference (6)). It is likely that the high abundance of MeCP2 in neurons is dependent to some extent on stability and the highly conserved amino acid sequence may serve to insure this. A measure of protein stability derived from gene ablation studies in adult mice suggested that MeCP2 has a half-life of ∼2 weeks in the brain (19), which is longer than average. A second generalization from studies of missense mutations is that disruption of the interactions between MeCP2 and DNA (R133C, T158M, T158A) or between MeCP2 and NCoR (R306C) is associated with RTT (13). It will be of interest to discover if modelling of additional RTT mutations in mice adds to this list of molecular causes. This information is likely to be essential in the search for rational therapeutic approaches to mutation-specific aetiologies underlying this condition.

## Materials and Methods

### Knock-in of *Mecp2* alleles

Targeting vectors to create EGFP-tagged alleles for WT *Mecp2*, *Mecp2R133C*, *Mecp2T158M* and *Mecp2R306C* were constructed using a 7.2-kb plasmid subclone of mouse 129/Ola genomic DNA (3), including *Mecp2* exons 3 and 4 (Supplementary Material, Fig. S1). The coding sequence of *EGFP* was fused in-frame to the end of the coding sequence of *Mecp2* in exon 4 followed by a *loxP*-flanked NeoStop cassette as a selectable marker, retaining the first 1.8 kb of the *Mecp2* 3′ UTR as the 3′ homology arm. Point mutations R133C, T158M and R306C were introduced into the WT *Mecp2-EGFP* targeting vector using the QuikChange II XL Site-Directed Mutagenesis Kit (Agilent Technologies). Linearized constructs were electroporated into 129/Ola E14 TG2a mouse ES cells and correctly targeted clones identified by PCR screening and Southern blotting (Supplementary Material, Fig. S1). Mice were generated from ES cells by standard procedures (3). The *loxP*-flanked selection cassette was either removed *in vitro* for ES cell differentiation into neurons by electroporation of targeted clones with pCAGGS-Cre plasmid, or *in vivo* by test mating chimeras with the CMV-Cre deleter strain. Mice have been submitted to Jackson Laboratories *T158M-GFP*: JAX Stock No. 26762 B6.Cg-*Mecp2*^tm4.1Bird^/J, *R133C-GFP*: JAX Stock No. 26848 B6.Cg-*Mecp2*^tm6.1Bird^/J and *R306C-GFP*:JAX Stock No. 26847 B6.Cg-*Mecp2*^tm5.1Bird^/J.

### RNA extraction and qPCR

Frozen half brains were homogenized in Tri Reagent (Sigma) using the Ultra-Turrax T25. RNA was extracted according to the manufacturer's instructions. RNA was DNase I treated (DNA-*free* kit, Ambion) and then reverse transcribed (iScript cDNA Synthesis Kit, Bio-Rad) according to the manufacturer's instructions. Fifty nanograms of cDNA was amplified in qPCR using SensiMix SYBR & Fluorescein Master Mix (Bioline) with the following primers: Me2Ex3/4 FWD ACCTTGCCTGAAGGTTGGAC, REV GCAATCAATTCTACTTTAGAGCGAAAA; CypA FWD TCGAGCTCTGAGCACTGGAG and REV CATTATGGCGTGTAAAGTCACCA. *Mecp2* mRNA was normalized to *Cyclophilin A* housekeeping gene expression. EGFP mutants were compared with WT littermates.

### Protein extraction and western blot

One frozen half brain was homogenized in ice-cold NE1 buffer [20 mm Hepes pH 7.9, 10 mm KCl, 1 mm MgCl_2_, 0.1% Triton X-100, 20% glycerol, 0.5 mm DTT, protease inhibitor (complete EDTA free cocktail, Roche)] before adding 750 units of benzonase for 15 min at room temperature then an equal volume of 2× SDS loading buffer (0.125 M Tris pH 6.8, 20% glycerol, 4% SDS, 0.25% bromophenol blue, 20 mm DTT, 0.3 M β-mercaptoethanol). Samples were boiled for 3 min before loading equal volumes on a 4–12% Run Blue SDS precast gel (Expedeon). Gels were transferred to a 0.2-μm nitrocellulose membrane (Bio-Rad) and then blocked for 1 h (5% milk, 1× TBS, 0.1% Tween) before applying anti-Mecp2 1:1000 (Sigma M6818) or anti-γ-tubulin 1:3000 (Sigma T5326) overnight at 4°C, followed by IRDye 800CW donkey anti-mouse IgG 1:10 000 (LiCor) for 2 h at RT after washing. Membranes were imaged using the Odyssey Infrared Imager (LiCor) and quantified using Image Studio Lite Software (LiCor).

### Phenotypic analysis

Mice tested were of fourth or fifth generation back-crossed to C57Bl/6J (N4 or N5). Scoring of symptoms and behavioural analyses were all performed blind to the genotype. Six parameters were examined at the same time each week (activity, gait, hindlimb clasping, tremor, breathing and general condition) and given a score between 0 and 2, as previously described (19). Animals that scored 2 for tremor, breathing or general condition, or which had lost 20% of their body weight had reached the severity limit of the experiment according to the Home Office License and were humanely culled. Mice were also weighed weekly. Behavioural analysis was done between 8 and 10 weeks in the order (1) elevated plus maze, (2) hanging wire and then (3) accelerating rotarod. For details of behavioural assays, see (19). In brief, for elevated plus maze, mice were placed in a cross-shaped maze 65 cm above the floor with 2 open arms (20 × 8 cm), 2 closed arms (20 × 8 × 25 cm) and a central area (8 × 8 cm) in uniform dim lighting. Time spent in open arms of the maze was visualized using ANY-maze software (Stoelting). For the hanging wire, time taken to bring one hind paw to a 1.5 mm diameter wire, 35 cm above the bench, after mice were suspended by forepaws was recorded for up to 30 s over 3 trials, inter-trial interval 15 min. For the accelerating rotarod, mice had one habituation training day then time taken to fall from a 3 cm diameter rotating rod, accelerating between 4 and 40 RPM over 5 min was recorded. Mice had 4 trials per day (inter-trial interval 1 h) over 3 days.

### Immunofluorescence

Mice were perfused with 3.7% paraformaldehyde in PBS, pH 7.4, then immersion fixed overnight. The brains were then dehydrated in 30% sucrose for 24 h, washed briefly in PBS, blotted dry, halved and snap frozen in isopentane cooled on dry ice. The brains were sectioned at 10 μm (Leica CM 1900 Cryostat) and mounted. Slides were washed in PBS, permeabilized in 0.1% Triton X-100 for 15 min, washed, blocked for 1 h at RT in 1.5% normal goat serum, stained with anti-NeuN-Cy3 1:100 (MAB377C3 Millipore) overnight at 4°C then finally, washed, stained with 1 μg/ml DAPI and washed again. Slides were mounted in Prolong Gold Antifade Reagent (Molecular Probes). The brains were visualized on a LeicaSP5 Confocal Laser Scanning Microscope. For clarity, a set of images were taken where the EGFP signal was optimized for each genotype individually. A look-up table (LUT) was applied to hippocampal CA1 images, where blue pixels denoted over-saturation and green pixels denoted zero. Photon multiplier tube (PMT) gain was kept constant, and laser power was adjusted until one or two pixels were blue and as few pixels as possible were green. These settings were saved and used for all subsequent images for that genotype.

### Electrophoretic mobility shift assays

Recombinant MeCP2 protein expression vectors were constructed with a C-terminal histidine tag in the bacterial expression plasmid pET30b (Novagen). The R133C point mutation was introduced into the WT plasmid using QuikChange II XL Site-Directed Mutagenesis Kit (Agilent Technologies) as per the manufacturer's instructions. BL21(DE3)pLysS competent cells were transformed, and a scrape of colonies was inoculated in a starter culture overnight, then into 500 ml warm LB (50 μg/ml kanamycin; 17 μg/ml chloramphenicol) and shaken at 37°C. Cultures were induced with 1 mm IPTG when the OD was 0.6–0.8 at 600 nm and then grown at 30°C for 3 h. Pellets were mashed in ice-cold lysis buffer [50 mm NaH2PO_4_, 100 mm NaCl, 10% glycerol, 30 mm imidazole, 0.1% NP40, protease inhibitor (complete EDTA free cocktail, Roche), pH 7.5] and passed through a 21G needle prior to adding 750 units of benzonase then being sonicated 30 s on/off for 10 cycles (Branson Digital Sonifier). Samples were adjusted to 0.3 M NaCl, and then after centrifugation, 0.5 ml bead volume NiSO_4_-coated Chelating Sepharose Fast Flow beads (GE Healthcare) in lysis buffer was added to lysates for 1 h at 4°C. Beads were washed 3 times in lysis buffer before MeCP2 polypeptides were eluted with 250 mm imidazole lysis buffer in 5 fractions. Eluted fractions were pooled, adjusted to 1 mm EDTA and then dialysed [Slide-A-Lyzer Dialysis Cassette 7 K MWCO (Thermo Scientific)] overnight at 4°C in 0.1 M HEPES buffer (20 mm HEPES pH7; 100 mm NaCl; 1 mm EDTA; 5 mm β-mercaptoethanol). The polypeptides underwent a second purification step and were eluted from a HiTrap SP HP 1 ml Column (GE Healthcare) using a 0.7 M NaCl HEPES buffer. An oligonucleotide probe from the mouse *Bdnf* promoter region 5′ AAGCATGCAATGCCCTGGAA***CG***GAATTCTTCTAATAAAAGATGTATCATTTTAAATGC 3′ (Biocore), plus complementary reverse strand, was methylated or unmethylated at the central CG indicated in bold italics. Five hundred nanograms of probe was radio-labelled using T4 polynucleotide kinase (NEB) and purified (MinElute PCR Purification Kit, Qiagen) according to the manufacturer's instructions. One nanogram probe and 1 μg poly deoxyadenylic-thymidylic acid competitor DNA (Sigma-Aldrich) were added to polypeptide in reaction buffer (5% glycerol, 0.1 mm EDTA, 10 mm Tris HCl pH7.5, 150 mm KCl, 0.1 mg/ml BSA) on ice for 20 min. Samples were run at 120 V for 70 min on a 10% acrylamide tris borate EDTA gel (0.075% APS, 0.00125% TEMED) in chilled TBE. The gels were exposed overnight and imaged using the Typhoon FLA 9500 scanner (GE Healthcare). Percentage shift was calculated using Image J software.

### Cellular immunoprecipitation

Using Lipofectamine 2000 (Life technologies), 1.5 × 10^6^ HEK 293 FT cells were transfected over night with 0.5 μg of MeCP2 WT or R133C, T158M and R306C mutants tagged with EGFP, according to the manufacturer's instructions. After assessment of transfection efficiency, the medium was changed and cells were transfected with annealed unmodified or methylated oligonucleotides (100 nM) final concentration using TransIT Oligofect reagent (Mirus) for 4 h. Cells were washed with PBS and harvested by trypsinisation. In the next step, cells were cross-linked with 1% formaldehyde for 5 min at room temperature and quenched by the addition of glycine to a final concentration of 0.125 M for 5 min. At this stage, cell pellets could be flash frozen in liquid nitrogen and stored at −80°C or directly used for DNA–protein complex isolation and consecutive IP. Oligonucleotide sequence: 5′ ATGCTAATTAACCCTCACTAAAGGGAACTCGAGACAT**CG**GAGAATTCACATCAC**CG**GTGAATCAGTGCTACC**CG**CAAGTGCACTGGATCCACTGGCCGTCGTTTTACAA 3′. The transfected fragment consisted of an artificial sequence (49% GC content) flanked by T3 and M13-20 primer binding sites for unique amplification. Primer sequences are underlined. Differentially methylated CGs are highlighted in bold. Soluble chromatin preparation and chromatin immunoprecipitation assays were carried out as described previously with modifications. In short, chromatin was sonicated using a Twin Bioruptor (Diagenode) 30 s on/off for 15 cycles. Equal amounts of chromatin were used for IP with 5 μg MeCP2 M6818 antibody (Sigma) and incubated overnight. Protein–antibody complexes were bound to magnetic protein G beads (Life technologies) for 4–5 h and washed with standard IP wash buffers for 10 min at 4°C. The cross-link was reversed by addition of 0.05 volume of 4 M NaCl over night at 65°C. After proteinase K digestion, DNA was recovered by phenol–chloroform–isoamylalcohol extraction and dissolved in 200 μl H_2_O. Real-time PCR of ChIP DNA and corresponding input DNA was performed using T3 and M13-20 primers.

### Brain chromatin immunoprecipitation

Frozen half brains were Dounce homogenized in 1 ml PBS and then fixed in 1% formaldehyde for 1 min at room temperature or 60 s after being on ice (stringent ChIP) before quenching with 250 mm glycine. Chromatin immunoprecipitation assays were carried out as described previously with modifications. In short, chromatin was sonicated using a Twin Bioruptor (Diagenode) 30 s on/off for 15 cycles. Equal amounts of chromatin were used for IP with 40 μl GFP-Trap_A beads (Chromotek) and rotated for 1.5 h at 4°C. Protein–antibody complexes were washed with standard IP wash buffers for 4 min at 4°C. The cross-link was reversed by addition of 0.05 volume of 4 M NaCl overnight at 65°C. After proteinase K digestion, DNA was recovered by phenol–chloroform–isoamylalcohol extraction and dissolved in 200 μl H_2_O. Real-time PCR of ChIP DNA and corresponding input DNA was performed using specific primers. Major Satellite FWD GGCGAGAAAACTGAAAATCACG, REV AGGTCCTTCAGTGTGCATTTC; LINE1 Elements FWD TTTGGGACACAATGAAAGCA, REV CTGCCGTCTACTCCTCTTGG; IAP FWD GAGATTGGACTTTTGACTTGT, REV TGTGGCTTGCTCATAGATTAG; *Bdnf* FWD TTCGATTCACGCAGTTGTTC, REV CTGAGCCAGTTACGTGACCA; Cyp3a25 FWD CAGGTTTGGGGTGTTGTGAA, REV CTGCAGCTGTTGTGGGAG; Cyp3a57 FWD GTGCTGCTCTTACATGGCTG, REV GTGGGGCTACAGTCTATGCT; Cyp3a59 FWD CCTGACTGGCTGCTCACTAT, REV AGGCTGTGAACTATAGGAGCC.

### Statistics

Behavioural data were analysed using the Kolmogorov–Smirnov test with the Benjamini and Hochberg method of correction for false discovery with multiple testing. Survival data were analysed using the Mantel-Cox test. Biochemical data were analysed using two-tailed, unpaired *t*-tests.

### Study approval

Mice were maintained under standard conditions and in accordance with UK Home Office regulations and licenses.

## Supplementary Material

Supplementary Material is available at *HMG* online.

## Funding

This work was supported by a consortium grant from the Rett Syndrome Research Trust, by a Wellcome Trust Edinburgh Clinical Academic Training studentship to Kyla Brown (100670), by a Wellcome Trust Programme Grant (091580) and by a Wellcome Trust Centre Core Grant (092076). S.L. was funded by a postdoctoral EMBO long-term fellowship (ALTF 1467-2011). Funding to pay the Open Access publication charges for this article was provided by The University of Edinburgh with support from The Wellcome Trust.

## Authors’ Contributions

K.B. and J.S. carried out mouse behaviour, biochemical analyses and immunohistochemistry. S.L. did cellular immunoprecipitation and qPCR validation of microarrays. S.L. and K.B. did ChIP. J.C. and K.B. did EMSAs. D.S., K.B. and M.K. did mouse phenotypic scoring. J.G. designed and engineered the targeting vector. J.S. and C.M. mutated targeting vectors. J.S., J.G., K.B. and C.M. did ES cell targeting. J.S. established all mouse lines. K.B. did neuronal differentiation and microarrays. J.S. and S.L. did FACS analysis. A.K. and S.W. analysed mouse behavioural data and microarray data, respectively. A.B. and K.B. produced the manuscript.

## Supplementary Material

Supplementary Data
