# Supplementary material for: The molecular basis of variable phenotypic severity among common missense mutations causing Rett syndrome
Source: Hum Mol Genet. 2015 Dec 8;25(3):558–70. doi: 10.1093/hmg/ddv496 (PMC4731022; doi:10.1093/hmg/ddv496)
Supplement: Supplementary Data [file supp_25_3_558__index.html]

The molecular basis of variable phenotypic severity among common missense mutations causing Rett syndrome — The molecular basis of variable phenotypic severity among common missense mutations causing Rett syndrome — Supplementary Data 

# The molecular basis of variable phenotypic severity among common missense mutations causing Rett syndrome

## Supplementary Data

Supplementary Data

- Supplementary Data - Pdf file
