# Supplementary material for: The molecular basis of variable phenotypic severity among common missense mutations causing Rett syndrome
Source: Hum Mol Genet. 2015 Dec 8;25(3):558–70. doi: 10.1093/hmg/ddv496 (PMC4731022; doi:10.1093/hmg/ddv496)
Supplement: Supplementary Data [file supp_ddv496_ddv496supp.pdf]

Supplementary Methods

Southern blot

ES cell DNA was digested with BamHI or KpnI (NEB) and resolved at 1.5V/cm on a 0.8% TAE agarose gel. Gels were treated with 0.25M HCl, then 0.4M NaOH before being transferred to a Zeta-Probe GT Nylon Blotting Membrane (Bio-Rad). Blots were washed in 2xSSC (300mM NaCl, 30mM Na citrate) before being blocked by modified Church & Gilbert hybridisation buffer (0.5M NaPi, 7% SDS, 1mM EDTA, 10mg/ml BSA, 50µg/ml herring sperm ssDNA (Sigma)) at 65°C. To assess the 5' end of the locus, DNA was probed with a 1.15kb HindIII fragment. To assess the 3' end of the locus, DNA was probed with a 1.1kb NcoI-BamHI fragment. Probes were labeled with P^{32} using the Megaprime DNA Labelling System (GE Healthcare) according to manufacturer's instructions, and hybridised overnight at 65°C. Blots were washed in 3xSSC and 1xSSC, before being visualised by phosphorimagery.

Neuronal differentiation

ES cells were plated in bacterial dishes (Greiner) in EB medium (GMEM (Gibco); 10% fetal bovine serum (Hyclone); 1mM sodium pyruvate; 1 x MEM non-essential amino acids; 50µM β-mercaptoethanol; 2mM L-glutamine (all Life Technologies)) on day 0 and the medium was changed every 2 days. 5µM retinoic acid was added to medium on day 4. On day 8, embryoid bodies were trypsinised, resuspended in N2 medium (DMEM/F12, 1 x N2 supplement, 1 x penicillin streptomycin (Invitrogen)), filtered (40µm cell strainer (BD Falcon)) and plated at 1.5 x 10^5 cells per cm^2 dish +/- coverslips. Dishes were pre-treated with 0.1mg/ml poly-DL-ornithine (Sigma) in 30mM boric acid, pH8.3 48hours and 2µg/ml laminin (Roche) 24 hours prior to

plating. Medium was gradually changed to Neurobasal with B27 supplement and penicillin streptomycin (Invitrogen) until neuronal cells were harvested six days after plating.

Neuronal immunofluorescence

Neuronal cells growing on coverslips were taken on day 6 post plating and fixed for 15 minutes with 4% paraformaldehyde pH 7.4 prior to staining with anti-NeuN-Cy3 1:100 (MAB377C3 Millipore) and 1μg/ml DAPI. Z stacks were taken using the same confocal settings.

Western blot

Neuronal cell western blot used anti-H3 antibody 1:50 000 (ab1791 Abcam) for the loading control. Cellular ChIP chromatin western blots used anti-GAPDH (D16H11 Cell Signaling) for the loading control.

FACS quantification

Mouse brain nuclei from wildtype MeCP2, T158M-, R133C- and R306C- GFP animals were prepared as previously described (39). After suspension in resuspension buffer (1x PBS supplemented with 20% glycerol and complete protease inhibitors (Roche), nuclei were washed twice in freshly prepared, sterile filtered PBTB buffer (1x PBS supplemented with 5% BSA, 0.1% Triton X-100 and complete protease inhibitors (Roche)) and passed through a 70μm cell strainer (Falcon). For NeuN co-staining, anti-NeuN-Cy3 antibody (MAB377C3 Millipore) was added in a 1:200 dilution and incubated in the dark for 30 minutes. Without washing, nuclei were analysed on FACSCalibur (BD) for EGFP and Cy3 expression.

Microarray gene expression

Frozen 6 week old male mouse cerebella were homogenized in Tri Reagent (Sigma) with the Ultra-Turrax T25 and RNA was extracted according to manufacturer's instructions, then enriched for mRNA with the RNeasy Mini Kit (Qiagen). Sample quality was verified using the Agilent 2100 Bioanalyzer and cRNA libraries prepared from 400ng using the Illumina Total Prep RNA Amplification Kit (Ambion), according to manufacturer's instructions. Biotinylated cRNA was assessed for quality then analysed on MouseWG-6 v2 BeadChips (Illumina) at the Wellcome Trust Clinical Research Facility (Edinburgh). Gene expression levels were analysed using the Bioconductor Limma package. Probes were removed from the analysis if they were detected in less than 3 samples and background correction, normalisation, transformation, differential expression analysis and annotation were performed on the remaining set following the protocol described in (42). For genes with multiple probes, mean log fold changes were calculated. Genes were ordered by length based on the Ensembl GRCm39 release 79 annotations and divided into bins using a window of 400 genes and a sliding step of 80 genes. The mean log fold change was plotted against the mean length of genes in each bin. qPCR validation primers are available on request.

Supplementary Figure 1

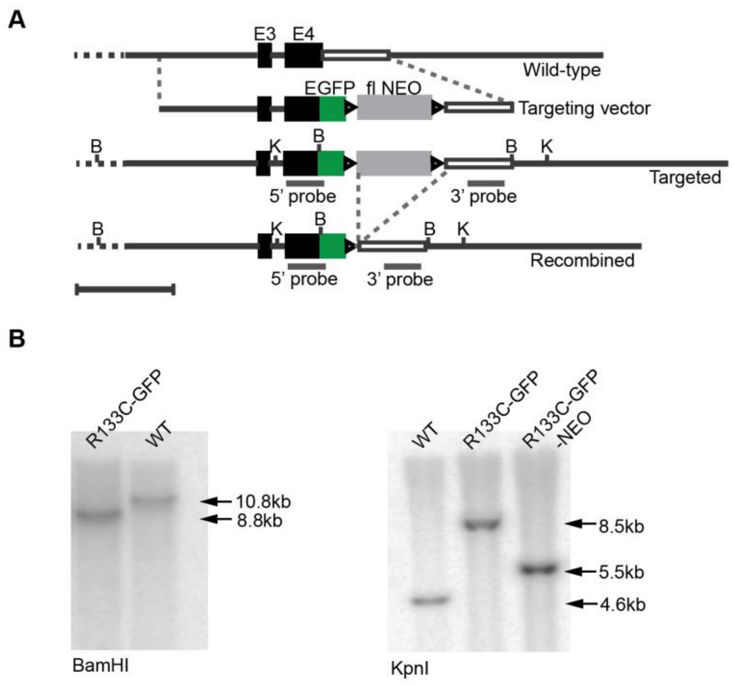

Brown et al Supplementary Figure 1

Design and creation of knock-in EGFP-tagged allelic series.

(A) Maps of the endogenous allele, targeting vector, targeted allele and recombined allele following exposure to CRE recombinase. E3, exon 3; E4, exon 4; EGFP, Enhanced Green Fluorescent Protein; fl NEO, floxed neomycin resistance cassette; B, BamHI restriction site; K, KpnI restriction site. Southern blot probes are indicated. White box represents 3' untranslated region. Scale bar 3kb. **(B)** Southern blot analysis of the targeted allele and targeted allele following CRE mediated recombination. *R133C-GFP* blots are shown as an example. Genomic DNA was probed following BamHI digest to check for homologous recombination at the 5' end of the locus (left). The 10.8kb band is seen in the *WT* allele, the 8.8kb band in the targeted allele.

Genomic DNA was probed following KpnI digest to check for homologous recombination at the 3’ end of the locus (right). The 4.6kb band is seen in the *WT* allele; 8.5kb in the targeted allele; and 5.5kb in the targeted allele, following CRE mediated recombination.

Supplementary Figure 2

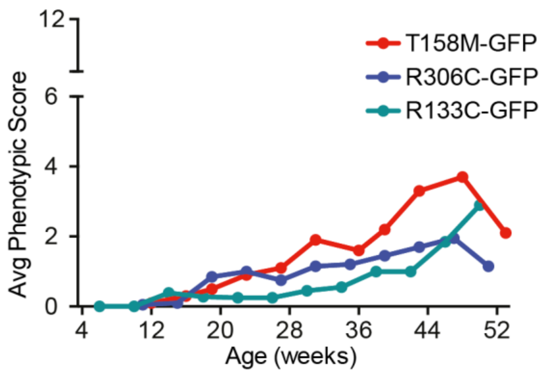

Brown et al Supplementary Figure 2

GFP allelic series heterozygous females display a milder RTT phenotype than the males and can be distinguished by average phenotypic score.

R133C-GFP (n=10), *R306C-GFP* (n=10) and *T158M-GFP* (n=14) female heterozygous mice were monitored for development of a RTT phenotype. Graph showing average phenotypic score over time. *T158M-GFP* and *R306C-GFP* mice underwent behavioural analysis at around 48 weeks, resulting in an improvement in their phenotypic score.

Supplementary Figure 3

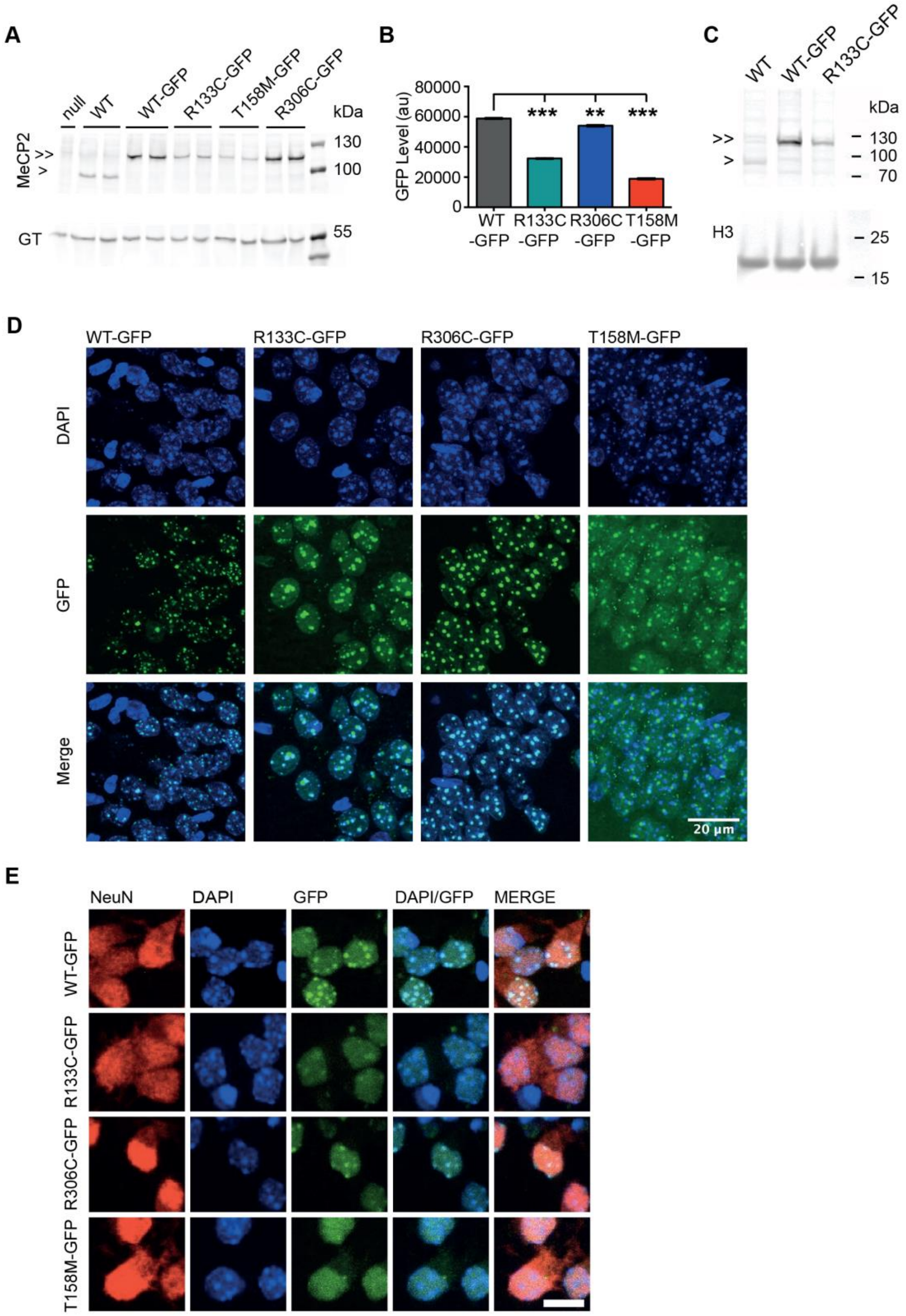

Brown et al Supplementary Figure 3

Reduced abundance and abnormal subnuclear localisation of R133C-GFP and T158M-GFP.

(A) Western blot of MeCP2-GFP abundance in the allelic series in relation to *Mecp2*-null and *WT* mouse brain. Gamma tubulin (GT) was a loading control. < MeCP2, << MeCP2-GFP. **(B)** Quantification of protein abundance for the allelic series using FACS and GFP detection verifies western blotting, n=3 biological replicates. Mean±SEM plotted. Statistical significance is denoted as follows: *p<0.05, **p<0.01, ***p<0.001 (unpaired two tailed t-Test). **(C)** Western blot from neuronal cells differentiated in vitro from an independent *R133C-GFP* ES cell clone showing reduced abundance of R133C-GFP relative to WT-GFP. < MeCP2, << MeCP2-GFP. Histone 3 (H3) was a loading control. **(D)** Representative images of the CA3 region of the hippocampus with confocal settings individualised for each genotype renders the abnormal patterns of subnuclear localisation in the MBD mutants more apparent. Scale bar 20μm. **(E)** Representative images of EGFP-tagged mutant neuronal cells differentiated in vitro. The MBD mutants have a mixed punctate and diffuse binding pattern. Cells were fixed and stained for DAPI (blue) and NeuN (red). Scale bar represents 10μm.

Supplementary Figure 4

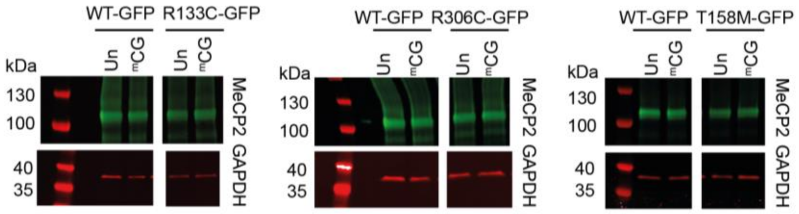

Brown et al Supplementary Figure 4

Equal MeCP2-GFP abundance between individual transfections in the cellular immunoprecipitation assay.

Western blots indicating an equal abundance of transfected protein for WT-GFP and the EGFP-tagged mutants in the cellular immunoprecipitation assay. Immunoprecipitated DNA and protein complexes corresponding to transfection of unmethylated (Un) and methylated (^{m}CG) oligonucleotides are shown.

Supplementary Figure 5

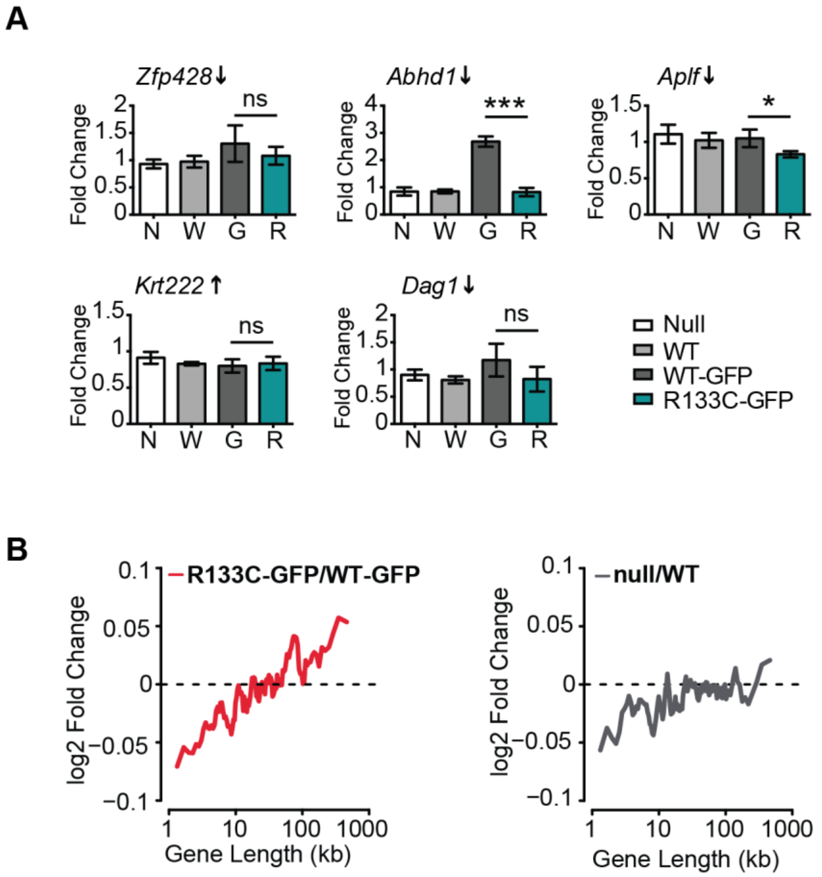

Brown et al Supplementary Figure 5

Modest upregulation of long genes in *R133C-GFP* cerebellum, relative to *WT-GFP*.

(A) Graphs showing qPCR for five of six genes that had significantly altered expression in *R133C-GFP* versus *WT-GFP* cerebellum by microarray. Significant change in expression was validated for 2 genes. N: *Mecp2*-null, W: WT, G: *WT-GFP*, R: *R133C-GFP*, n=3 biological replicates. Mean fold change relative to *Cyclophilin A* housekeeping gene expression ± SD plotted. Statistical significance is denoted as follows: *p<0.05, **p<0.01, ***p<0.001 (unpaired, two-tailed t-Test). Arrows denote

the direction of change in *R133C-GFP* cerebellum relative to *WT-GFP* cerebellum observed in the microarray analysis. **(B)** Graph plotting gene expression relative to gene length for *R133C-GFP* compared to *WT-GFP* cerebellum (left) and *Mecp2*-null compared to *WT* cerebellum. n=3 biological replicates.
